# The *WUSCHEL‐RELATED HOMEOBOX 3* gene *PaWOX3* regulates lateral organ formation in Norway spruce

**DOI:** 10.1111/nph.13536

**Published:** 2015-06-25

**Authors:** José M. Alvarez, Joel Sohlberg, Peter Engström, Tianqing Zhu, Marie Englund, Panagiotis N. Moschou, Sara von Arnold

**Affiliations:** ^1^ Department of Plant BiologyUppsala BioCenterSwedish University of Agricultural Sciences, and the Linnean Center for Plant BiologyPO Box 7080SE‐75007UppsalaSweden; ^2^ Department of Organismal BiologyPhysiological BotanyUppsala University, and the Linnean Center for Plant BiologyPO Box 7080SE‐75007UppsalaSweden

**Keywords:** cotyledon, embryogenesis, lateral organ, needle, Norway spruce (*Picea abies*), *WUSCHEL‐RELATED HOMEOBOX*

## Abstract

- In angiosperms, *WUSCHEL‐RELATED HOMEOBOX 3* (*WOX3*) genes are required for the recruitment of founder cells from the lateral domains of shoot meristems that form lateral regions of leaves. However, the regulation of the formation of lateral organs in gymnosperms remains unknown.
- By using somatic embryos of Norway spruce (*Picea abies*) we have studied the expression and function of *PaWOX3* during embryo development. The mRNA abundance of *PaWOX3* was determined by quantitative real‐time PCR, and the spatial expression of *PaWOX3* was analysed by histochemical β*‐*glucuronidase (GUS) assays and *in situ* mRNA hybridization. To investigate the function of *PaWOX3*, we analysed how downregulation of *PaWOX3* in RNA interference lines affected embryo development and morphology.
- *PaWOX3* was highly expressed in mature embryos at the base of each cotyledon close to the junction between the cotyledons, and in the lateral margins of cotyledons and needles, separating them into an adaxial and an abaxial side. Downregulation of the expression of *PaWOX3* caused defects in lateral margin outgrowth in cotyledons and needles, and reduced root elongation.
- Our data suggest that the *WOX3* function in margin outgrowth in lateral organs is conserved among the seed plants, whereas its function in root elongation may be unique to gymnosperms.

## Introduction

In seed plants major patterning events, including the establishment of stem cell niches in shoot and root meristems, take place during embryogenesis. Through the activities of stem cells, plants can produce new organs throughout their lifetimes. Lateral organs are initiated at the periphery of the shoot apical meristem (SAM) by recruitment of a group of founder cells (Poethig & Szymkowiak, 1995). Arabidopsis (*Arabidopsis thaliana*) has widely been used as a model organism for studying developmental patterning in plants and especially embryogenesis, where a stereotyped cell division pattern makes it possible to follow the fate of different cells from the early embryo (Laux *et al*., 2004). This regularity has been used to identify the origin of developmental defects in embryo‐defective mutants in Arabidopsis (Jürgens *et al*., 1991). However, embryogenesis in Arabidopsis is not representative for all plant species. Although the basic body organization of the seedling is similar in different higher plant species, its developmental origin is taxa‐dependent. Therefore, evolutionary developmental biology approaches using other models are needed for comparisons, for example between gymnosperms and angiosperms, which share a common ancestor *c*. 300 Myr ago (Savard *et al*., 1994; Smith *et al*., 2010). The regulation of embryo development in gymnosperms is poorly understood compared with angiosperms, partly owing to the lack of characterized embryo‐defective mutants. However, by using somatic embryos and reverse genetics it has been possible to study the regulation of embryo development in some conifers (von Arnold & Clapham, 2008).

In angiosperms, members of the *WUSCHEL‐RELATED HOMEOBOX* (*WOX*) gene family of transcription factors play important roles in the cell‐fate determination during plant development. Phylogenetic analyses have identified three major clades in the *WOX* gene family: the modern clade/WUS clade, specific to seed plants; the intermediate clade, present in vascular plants; and the ancient clade, with representatives in the earliest diverging green plants and therefore probably representing an ancestral *WOX* gene (van der Graaff *et al*., 2009). Thus, the major diversification within the *WOX* gene family took place before the gymnosperm–angiosperm split *c*. 300 Myr ago. We have previously identified 11 *WOX* genes in Norway spruce (*Picea abies*), which are phylogenetically interspersed in the three clades of the angiosperm *WOX* gene family (Hedman *et al*., 2013). One gene, *PaWOX13*, was placed in the ancient clade, five genes (*PaWOX8/9*,* PaWOX8A‐D*) were found to group in a gymnosperm‐specific clade within the intermediate clade, and five genes (*PaWUS*,* PaWOX2*,* PaWOX3*,* PaWOX4* and *PaWOX5*) grouped with the modern clade.

Functional information of gymnosperm *WOX* genes is, to our knowledge, available only for the Norway spruce *PaWOX8/9* gene, which is expressed in early embryos and is homologous to both *AtWOX8* and *AtWOX9* (Palovaara *et al*., 2010; Hedman *et al*., 2013). We have found that *PaWOX8/9* is required for the correct orientation of the cell division plane and cell fate determination during early embryo development (Zhu *et al*., 2014), in accordance with what previously has been shown in Arabidopsis (Haecker *et al*., 2004; Breuninger *et al*., 2008). This suggests that *PaWOX8/9* performs an evolutionarily conserved function as a regulator of the establishment of the apical–basal embryo pattern.

In Arabidopsis, the stem‐cell regulating *WOX* genes *AtWUS* and *AtWOX5* are involved in the maintenance of the SAM and root apical meristem (RAM), respectively (Mayer *et al*., 1998; Sarkar *et al*., 2007). However, the shoot‐specific expression of *WUS* and root‐specific expression of *WOX5* seem to be specific to angiosperms. Nardmann *et al*. (2009) identified single homologues of *WUS*/*WOX5* in three gymnosperms which were expressed in both the shoot and the root, suggesting that *WUS* and *WOX5* were derived from a gene duplication in the angiosperm lineage. The functional divergence between these genes appears to have resulted primarily from the evolution of divergent expression patterns, whereas the molecular function of the gene product is highly conserved for WUS/WOX5 (Sarkar *et al*., 2007) and WUS/WOX3 (Shimizu *et al*., 2009), and partially conserved for WOX3/WOX4 (Ji *et al*., 2010).

In angiosperms, a functional conservation between dicots and monocots has been shown for the *WOX3* clade that includes, among others, the *PRESSED FLOWER* (*PRS*) gene in Arabidopsis, the duplicated genes *NARROW SHEATH1* and *2* (*NS1/NS2*) in maize (*Zea mays*), and the duplicated genes *NARROW LEAF2* and *3* (*NAL2*/*NAL3*) in rice (*Oryza sativa*). *PRS* in Arabidopsis and *NS1/NS2* in maize are important for the recruitment of founder cells from lateral domains of SAMs and lateral outgrowth of leaves (Scanlon *et al*., 1996; Matsumoto & Okada, 2001; Nardmann *et al*., 2004; Shimizu *et al*., 2009). *NAL2*/*NAL3* in rice regulate leaf width (Ishiwata *et al*., 2013). *AtWOX1*, another *WOX* gene belonging to the *WOX3* clade, which is expressed in the middle domain between the adaxial and abaxial domains in leaves in Arabidopsis (Nakata & Okada, 2012), acts redundantly with *PRS* and is important for lateral‐specific blade outgrowth and margin‐specific cell fate (Nakata *et al*., 2012).

In gymnosperms, single orthologues of *WOX3* have been identified, whereas *WOX1* orthologues have not been found (Hedman *et al*., 2013; Nardmann & Werr, 2013). *WOX3* expression in Scots pine (*Pinus sylvestris*) is initially found in a few cells at the surface of the SAM periphery and later in apical and marginal initials of lateral organs, suggesting a common ancestry of *WOX3* in gymno‐ and angiosperms which predetermines positioning of the incipient leaf primordium (Nardmann & Werr, 2013).

In this report, we show that *PaWOX3* has an expression pattern which is similar to the corresponding orthologue in pine in the shoot apex. We further provide direct genetic evidence that *PaWOX3* is a regulator of margin outgrowth in lateral organs, suggesting an evolutionarily conserved function of *WOX3* among seed plants. In addition, *PaWOX3* has a specific role in the regulation of root elongation, which might be unique for gymnosperms.

## Materials and Methods

### Plant material

The embryogenic line 61 : 21 of Norway spruce (*Picea abies* L. Karst) was grown as described previously (von Arnold & Clapham, 2008). Briefly, proembryogenic masses (PEMs) were cultured on solidified proliferation medium containing 9 μM 2,4‐dichlorophenoxyacetic acid (2,4‐D) and 4.5 μM benzyladenine (BA) as plant growth regulators (PGRs). To stimulate differentiation of early embryos (EEs) the cultures were transferred to prematuration medium lacking PGRs for 1 wk. Thereafter, the cultures were transferred to maturation medium containing 30 μM abscisic acid (ABA) for development of late embryos (LEs) and mature embryos (MEs). MEs were desiccated for 1 wk and then germinated for up to 4 months.

Adventitious buds were induced by culturing MEs on bud induction medium (germination medium containing 10 μM BA) for 7 d and then transferred to germination medium to allow development of adventitious buds.

For *in situ* hybridization, vegetative buds from adult Norway spruce trees were collected in October (bud dormancy) and April (active growth).

### RNA extraction, cDNA synthesis and quantitative real‐time PCR

In order to analyse the mRNA abundance of *PaWOX3* (accession number JX411947) during embryo development, samples at different developmental stages including PEMs, EEs, early late embryos (LE1s), late embryos (LE2s), maturing embryos (ME1s), fully matured embryos (ME2s) and germinated embryos after 1 (G1), 2 (G2), 3 (G3), and 6 (G6) wk on germination medium were collected. Cotyledons, needles, shoot tips (SAM with 4–5 needle primordia covered by bud scales), root tips and lateral roots were collected from 16‐wk‐old plants. To analyse the mRNA abundance of *PaWOX3* in developing adventitious buds, 50 embryos treated or nontreated with BA were collected 1, 2, 3 and 6 wk after the start of bud‐induction treatment. Batches of 100 mg tissue for each sample were frozen in liquid nitrogen and stored at −80°C until use.

The samples were ground in liquid nitrogen and total RNA was extracted using the Spectrum Plant Total RNA kit (Sigma‐Aldrich) following the manufacturer's protocol. RNA concentration was estimated using a nanodrop spectrophotometer (Thermo Scientific, Waltham, MA, USA) and the RNA integrity was tested in a 1% (w : v) agarose gel. One microgram of total RNA was reverse transcribed using the RevertAid H Minus First Strand cDNA Synthesis Kit (Fermentas, Thermo Scientific, Helsingborg, Sweden) following the manufacturer's protocol.

The quantitative real‐time (qRT)‐PCR analysis was performed on a Bio‐Rad iCycler iQ PCR Thermal Cycler using the iQ5 Real‐Time Detection System and 96‐well PCR plates sealed with adhesive seals (Bio‐Rad). Approximately 10 ng cDNA was used per well. Amplifications were performed using the following protocol: 95°C for 10 min, 45 cycles at 95°C for 10 s and at 60°C for 30 s. RT‐PCR specificity was assessed using negative controls (no template), RT‐control (nonreverse transcribed RNA), a melting curve analysis and by gel electrophoresis of a group of selected reactions. The mRNA abundance of *PaWOX3* was normalized to the mRNA abundance of *ELONGATION FACTOR 1* (*PaEF1*) (Hedman *et al*., 2013). Primer sequences are presented in Supporting Information Table S1. Three biological replicates, each with three technical replicates, were assayed for each different embryo developmental stage. Significant differences in mRNA levels were determined by *t*‐test analysis or ANOVA using the Student–Newman–Keuls test for *post hoc* comparisons (sigmaplot v11 software, Chicago, IL, USA).

### Vector construction and genetic transformation

In order to obtain RNA interference (RNAi), two fragments of 333 and 281 bp that overlap in the 3′ region of the *PaWOX3* coding sequence (CDS) were amplified and fused to form a hairpin structure (Fig. S1). *Eco*RI and *Bam*HI restriction sites were added on forward primers of each fragment as a linker (Table S1). The hairpin was confirmed by sequencing, subcloned into pENTR^™^/D‐TOPO^®^ (Invitrogen) and then transferred by *att* site LR recombination into the destination vector pMDC32 (2×35S promoter) (Curtis & Grossniklaus, 2003). The resulting vector was designated as *PaWOX3i*.

The upstream sequence of *PaWOX3* was obtained using the GenomeWalker Kit (Clontech, Palo Alto, CA, USA), following the manufacturer's instructions. A fragment of 2025 bp upstream of the CDS of *PaWOX3* was amplified from genomic DNA. The upstream fragment was first subcloned into pENTR^™^/D‐TOPO^®^ (Invitrogen) and then transferred by *att* site LR recombination into the destination vector pGWB3 to drive the expression of the the β*‐*glucuronidase (GUS) reporter gene (Wheeler *et al*., 2008). The resulting vector was designated as *pPaWOX3:GUS*.

Vectors were introduced by electroporation into the *Agrobacterium tumefaciens* C58C1 strain carrying the additional virulence plasmid pTOK47. A pMDC32‐*GUS* (2x35S:*GUS*) vector was used as transformation control.

Norway spruce embryogenic cultures were transformed by cocultivation with *A. tumefaciens* as previously described in Zhu *et al*. (2014). Single putative stable transformants were grown on selection medium for at least 4 wk. DNA from somatic embryos was extracted using the DNeasy Plant Mini Kit (Qiagen) following the manufacturer's instructions. Putative transformants were PCR‐tested and the PCR products were sequenced. Downregulation was checked in ME2s from RNAi lines by qRT‐PCR. The GUS activity in reporter lines was analysed histochemically according to Jefferson *et al*. (1987). GUS staining was analysed after 72 h incubation at 37°C in GUS solution, unless otherwise stated. When needed, samples were bleached in a hydrogen peroxide (H_2_O_2_): acetic acid (1 : 1) solution at 90°C for 30 min and then cleared in a 2.5 g ml^−1^ chloral hydrate solution at 4°C for at least 12 h after the GUS staining. Based on qRT‐PCR and GUS activity results, four out of 24 selected *PaWOX3i* lines (*PaWOX3i.3*,* PaWOX3i.13*,* PaWOX3i.18* and *PaWOX3i.24*) and three out of 12 selected reporter lines (*pPaWOX3:GUS.1*,* pPaWOX3:GUS.2*, and *pPaWOX3:GUS.6*), as well as the 61 : 21 line as untransformed control (U‐control) and a 2x35S:*GUS* line as transformed control (T‐control) were chosen for further analysis.

### Morphological analyses in RNAi lines

In order to assess the effect of *PaWOX3* on embryo maturation, 50 LE1s per control and RNAi line were subjected to time‐lapse tracking analysis for 12 d.

Mature embryos of each control and *PaWOX3i* line were transferred to germination medium. The number of cotyledons per embryo was recorded in mature embryos at the time of transfer to germination medium. The frequency of aberrant cotyledons was calculated after 6 and 12 wk. Young plants were potted and cultured in a glasshouse for 12 wk at 20°C and a 16 h : 8 h photoperiod to allow shoot growth.

The effect of *PaWOX3* on radicle emergence (germination frequency) was estimated after 4 wk on germination medium. Root elongation and number of lateral roots were recorded after 16 wk on germination medium.

All samples were examined under a Leica MZFL III stereomicroscope (Leitz, Germany) or a Zeiss Axioplan microscope and micrographs were acquired with a DFC490 camera.

### RNA *in situ* hybridization (ISH)

For RNA ISH the following materials were used: ME2s, shoot tips collected after 6 wk of germination and vegetative buds from adult trees collected in October and April. *In situ* hybridization was performed essentially as described by Jackson (1991) and Tandre *et al*. (1998). Sections of 7 μm were hybridized to 2 ng ^35^S‐labelled RNA probes. A gene‐specific fragment was used as probe. The probes were obtained as described in Englund *et al*. (2011) (see primer sequences in Table S1). The slides were coated with NBT2 emulsion (Eastman Kodak, Rochester, NY, USA) and exposed for *c*. 8 wk. The sections were photographed using a Leica microscope equipped with a Leica DFC490 camera and the pictures were processed using Adobe Photoshop CS6 13.0 software (San Jose, CA, USA).

## Results

### Expression pattern of *PaWOX3*

**Figure 1:**
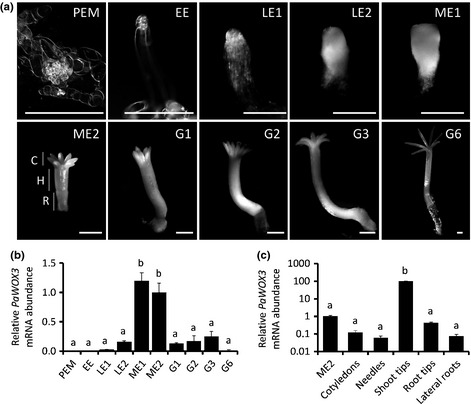
Quantitative real‐time (qRT)‐PCR analysis of the *PaWOX3* mRNA abundance in Norway spruce (*Picea abies*) during the development and germination of somatic embryos, and in different parts of young plants. (a) Embryo developmental stages used for the qRT‐PCR analysis. Proliferating proembryogenic mass (PEM) in the presence of the plant growth regulators (PGRs) auxin and cytokinin; early embryo (EE) 1 wk after withdrawal of PGRs; early late embryo (LE1) and late embryo before the emergence of cotyledons (LE2) after 1 and 2 wk on the maturation medium, respectively; maturing embryo (ME1) and fully matured embryo (ME2) after 4 and 6 wk on the maturation medium, respectively. Embryos 1 (G1), 2 (G2), 3 (G3), and 6 (G6) wk after transfer to germination medium. C, cotyledon; H, hypocotyl; R, root cap. Note that the number of cotyledons in Norway spruce varies between 6 and 11. Bars, 1 mm. (b) Relative *PaWOX3* mRNA abundance during development and germination of somatic embryos in the different stages described in (a). (c) Relative *PaWOX3* mRNA abundance in cotyledons, needles, shoot tips, root tips and lateral roots of 16‐wk‐old plants. The mRNA abundances in (b) and (c) are relative to the mRNA level in ME2 and normalized against *PaEF1*. The mRNA abundances are means ± SE of three biological replicates with three technical replicates each. Different letters indicate significant differences in the relative *PaWOX3* mRNA abundance (Student–Newman–Keuls test, α  =  0.05).

The development of Norway spruce somatic embryos has been described previously (Filonova *et al*., 2000; Larsson *et al*., 2012). The developmental stages analysed in this study include proliferating PEM, EE, early late embryo (LE1), late embryo (LE2), maturing embryo (ME1) and fully matured embryo (ME2), as well as germinated embryos after 1 (G1), 2 (G2), 3 (G3) and 6 (G6) wk on germination medium (Fig. 1a).

The qRT‐PCR data (Fig. 1b) showed that the mRNA level of *PaWOX3* was below the detection threshold in PEMs and EEs, low in LE1s and LE2s but increased significantly in mature embryos (ME1s and ME2s). The mRNA abundance of *PaWOX3* declined during germination. The peak of mRNA abundance detected in ME1 and ME2 suggests that *PaWOX3* could have a role in the formation of SAM, RAM and/or the emergence of cotyledons, the main processes occurring during these stages (Filonova *et al*., 2000). In addition, *PaWOX3* mRNA was detected in cotyledons, needles, shoot tips, root tips and lateral roots from 16‐wk‐old plants, with the highest level found in shoot tips (Fig. 1c).

**Figure 2:**
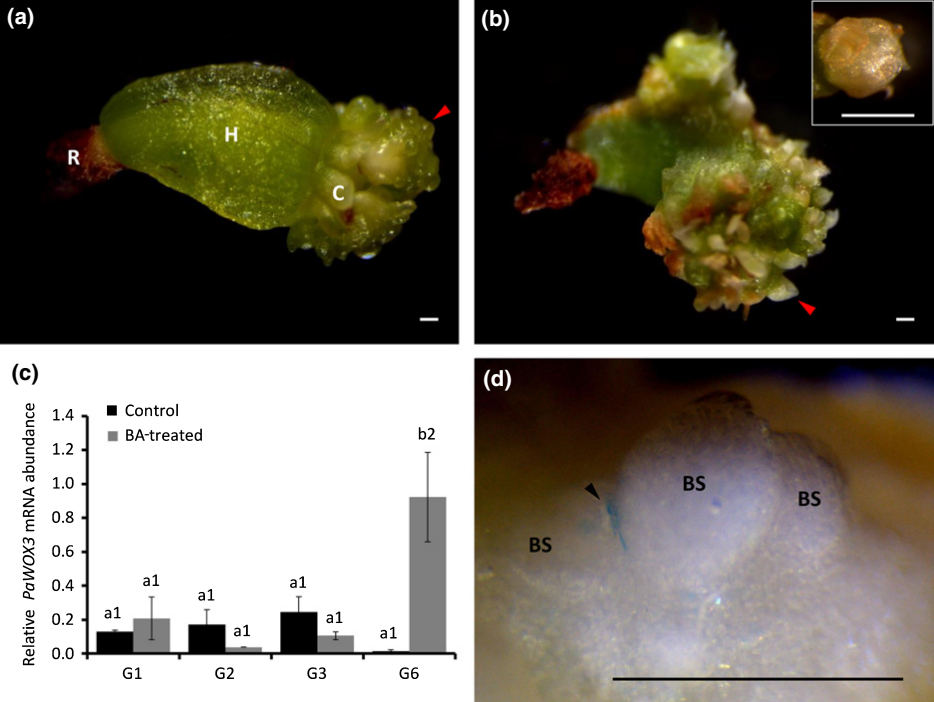
Adventitious buds on benzyladenine (BA)‐treated embryos of Norway spruce (*Picea abies*). (a) BA‐treated embryo after 3 wk (G3). C, cotyledon; H, hypocotyl; R, root cap. Note the emergence of meristemoids (red arrowhead). (b) BA‐treated embryo after 6 wk (G6). Note the well‐developed bud covered by bud scales (red arrowhead and inset). (c) Relative *PaWOX3* mRNA abundance in BA‐treated and nontreated embryos after 1 (G1), 2 (G2), 3 (G3) and 6 (G6) wk. The mRNA levels are relative to the mRNA level in mature embryos (ME2) not treated with BA and normalized against *PaEF1*. The mRNA levels are means ± SE of three biological replicates with three technical replicates each. Different letters indicate significant differences in the relative *PaWOX3* mRNA abundance between control and BA‐treated embryos at the same developmental stage (Student–Newman–Keuls test, α  =  0.05). Different numbers indicate significant differences in the relative *PaWOX3* mRNA abundance between the different developmental stages in control and BA‐treated embryos separately (Student–Newman–Keuls test, α  =  0.05). (d) Adventitious bud, developed on 12‐wk‐old BA‐treated *pPaWOX3:GUS* embryo, with bud scales (BS) covering the shoot apical meristem. Note the β*‐*glucuronidase (GUS) signal at the base of the bud scale on the left that was pushed aside after GUS staining (black arrowhead). Bars, 0.5 mm.

Adventitious buds were induced by treating ME2s with BA for 1 wk (von Arnold & Hawes, 1989). Meristemoids developed on all embryos during the second week after the BA treatment (Fig. 2a). These meristematic regions developed further into adventitious buds covered by bud scales (Fig. 2b). The mRNA abundance of *PaWOX3* was similar in nontreated and BA‐treated embryos during the first 3 wk. However, when adventitious buds had developed in G6 embryos the mRNA level of *PaWOX3* was significantly higher in BA‐treated embryos (Fig. 2c).

**Figure 3:**
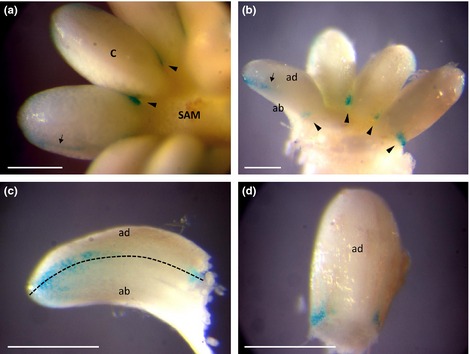
Expression pattern of *PaWOX3* in mature embryos of Norway spruce (*Picea abies*) according to β*‐*glucuronidase (GUS) assay results. GUS signal was developed in *pPaWOX3:GUS* lines after incubation in GUS solution at 37°C for 72 h. (a) GUS staining at the base of cotyledons between neighbour cotyledons (black arrowheads) and in the lateral margins dividing the cotyledon into an adaxial and an abaxial side (black arrow) in a mature embryo viewed from the top and (b) after removal of some of the cotyledons. (c) Detail of a cotyledon viewed from the side. Dashed line, hypothetical ad/abaxial axis. (d) Detail of a cotyledon viewed from the base‐top. C, cotyledon; SAM, shoot apical meristem; ad, adaxial side; ab, abaxial side. Bars, 0.5 mm.

**Figure 4:**
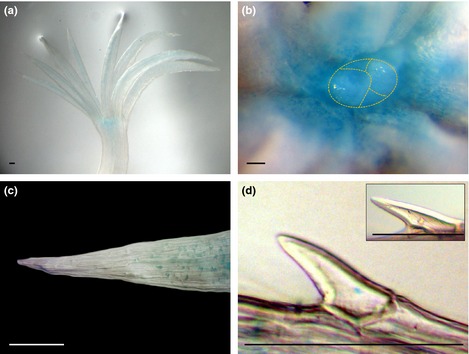
Expression pattern of *PaWOX3* in 6 wk‐old plants of Norway spruce (*Picea abies*) according to β*‐*glucuronidase (GUS) assay analyses. (a) Shoot tip. (b) Detail of the epicotyl. Note the GUS signal in the bud scales (yellow dashed line) and the base of cotyledons. (c) Detail of a cotyledon tip. Note the lack of GUS signal in the most apical part. (d) Detail of a sawtooth hair on a cotyledon from a *pPaWOX3:GUS* plant and a control plant (inset). Note the GUS signal inside the sawtooth hair from *pPaWOX3:GUS* plant. Bars, 0.2 mm.

In order to gain more insight into the spatial expression of *PaWOX3*, histochemical GUS assays on mature *pPaWOX3:GUS* embryos and young plants were performed. GUS staining in mature *pPaWOX3:GUS* embryos was detected at the base of the cotyledons close to the junction between cotyledons, and in the lateral margins dividing the cotyledon into an adaxial and an abaxial side (Fig. 3). GUS staining could not be detected in the SAM. A weak GUS signal was observed at the base of the bud scales covering adventitious buds on *pPaWOX3:GUS* embryos (Fig. 2d), but not in the central zone of the SAM. These results support the notion that *PaWOX3* function is associated with the emergence of lateral organs but not with meristem formation. A strong GUS staining was observed in the basal part of mature embryos, whereas the mRNA level of *PaWOX3* was low (Notes S1). This discrepancy might indicate that the GUS expression in the basal part of the embryo is not reliable. In 6‐wk‐old plants, weak GUS signals were detected in cotyledons (Fig. 4a) and bud scales covering the SAM (Fig. 4b). When the bud scales were removed before GUS staining, no signal could be detected in the SAM. The weak GUS staining was observed in a patchy pattern all around the cotyledons except at the tip (Fig. 4c). It has not been possible to trace the patchy expression pattern to any specific cell type. GUS signal was also detected in the sawtooth hairs on the cotyledon margins (Fig. 4d). Although expression of *PaWOX3* was detected in roots by qRT‐PCR, GUS staining was not detectable in roots either before or after bleaching and clearing.

**Figure 5:**
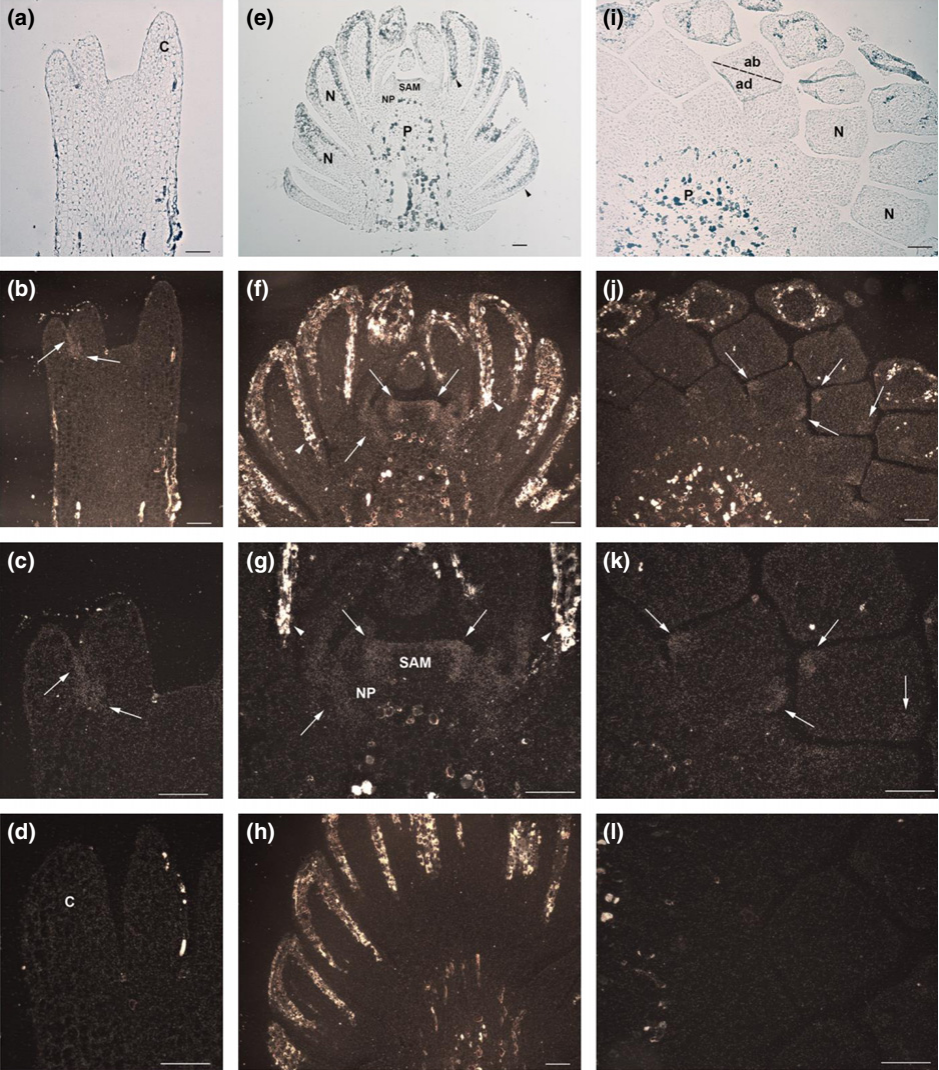
Expression pattern of *PaWOX3* in mature embryos and adult vegetative buds of Norway spruce (*Picea abies*) according to mRNA *in situ* localization results. Hybridization signals appear as white grains (white arrows) in dark field microscopy. Dark areas from phenolic compounds produce white reflections in the dark field images (black and white arrowheads). (a–d) Longitudinal section of mature embryos. (a) Antisense probe in bright field. (b, c) Antisense probe in dark field. Note the signal at the base and border of cotyledons. (d) Sense probe in dark field. (e–h) Longitudinal sections of adult vegetative buds collected in April. (e) Antisense probe in bright field. (f, g) Antisense probe in dark field. Note the signal in the laterals of shoot apical meristems (SAM) and in needle primordia. (h) Sense probe in dark field. (i–l) Cross‐sections of adult vegetative buds collected in April. (i) Antisense probe in bright field. (j, k) Antisense probe in dark field. Note the signal in two opposite poles in needles. (l) Sense probe in dark field. C, cotyledon; NP, needle primordium; N, needle; P, pith; ab, abaxial side; ad, adaxial side. Dashed line, hypothetical ad/abaxial axis. Bars, 0.1 mm.

Results obtained by GUS staining and qRT‐PCR analyses of the apical part of the mature embryos, were in good agreement with those obtained by *in situ* mRNA hybridization. *PaWOX3* mRNA in mature embryos was detected at the base of the cotyledons, and in the lateral margins dividing the cotyledon into an adaxial and an abaxial side (Fig. 5a–d). No signals were detected in the basal part of the mature embryos, likely due to the very low mRNA level of *PaWOX3* in this part of the embryo (Notes S1). *In situ* mRNA localization analyses were also conducted on adult vegetative buds collected during dormancy in autumn and at the time of shoot elongation in spring. No signal was detected in resting buds collected in October. In growing buds collected in April, the mRNA was detected primarily in the lateral parts of the shoot meristem, and in needle primordia (Fig. 5e–h). In cross‐sections of needles, distinct hybridization signals were detected in two opposite poles, corresponding to the lateral margins of the needles, separating its ad‐ and abaxial sides (Fig. 5i–l).

### *PaWOX3* is required for normal development of cotyledons and needles

**Figure 6:**
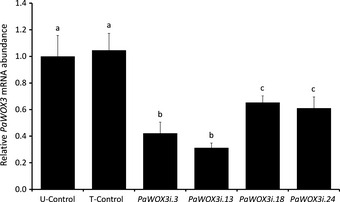
Quantitative real‐time PCR analysis of the relative *PaWOX3* mRNA abundance in mature embryos (ME2) of Norway spruce *(Picea abies)* from control (U‐control and T‐control) and *PaWOX3i* lines. mRNA levels are relative to the mRNA level in the U‐control and normalized against *PaEF1*. The mRNA abundances are means ± SE of three biological replicates with three technical replicates each. Different letters indicate significant differences in the relative *PaWOX3* mRNA abundance among lines (Student–Newman–Keuls test, α  =  0.05).

In order to establish the function of *PaWOX3* during embryo development and germination, RNAi lines for *PaWOX3* were constructed. The qRT‐PCR data showed that the mRNA level in most of the 24 *PaWOX3i* lines obtained was significantly decreased and in some of the lines by > 60% compared with the mRNA abundance in the corresponding untransformed (U‐control) and transformed (T‐control) controls. Four RNAi lines were selected for further studies: *PaWOX3i.3* and *PaWOX3i.13* with a strong downregulation, and *PaWOX3i.18* and *PaWOX3i.24* with intermediate levels of downregulation (Fig. 6).

In a time‐lapse tracking analysis of 50 LE1s from each of the four *PaWOX3* RNAi lines and the two controls (U‐control and T‐control) three developmental pathways were observed: normal embryo maturation; formation of ball‐shaped embryos, in which the embryos lacked differentiated cotyledons; and embryo degeneration, in which embryogenic tissue differentiated from the first selected embryo (Fig. S2). The frequency of LE1s following the different developmental pathways were similar in the *PaWOX3* RNAi lines as in the T‐control, which suggests that a reduction in *PaWOX3* expression does not affect the development of MEs.

**Figure 7:**
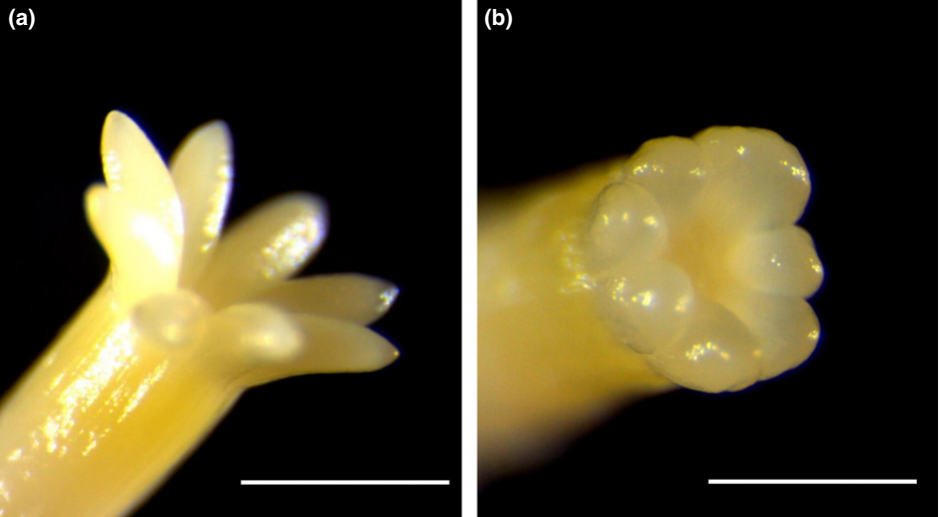
Cotyledon morphology in mature embryos of Norway spruce *(Picea abies)*. (a) Phenotype of a mature embryo from a control line. (b) Typical phenotype of a mature embryo from *PaWOX3i* lines (the embryo shown is from line *PaWOX3i*.24). Note that the cotyledons in the embryo from the *PaWOX3i* line are shorter, thicker, have a less pointed tip and a less defined adaxial/abaxial side than the control embryos. Bars, 1 mm.

Cotyledons in mature embryos from *PaWOX3i* lines were usually shorter, thicker and had a less pointed tip than those from control lines (Fig. 7). The number of cotyledons per embryo varied between 6 and 11 in all lines. No significant differences were found in the average number of cotyledons per embryo between control and *PaWOX3i* lines (Fig. S3).

**Figure 8:**
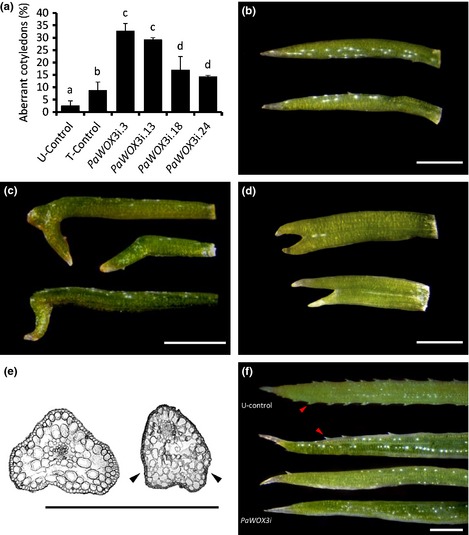
Abnormalities in cotyledons and needles in Norway spruce *(Picea abies)* plants from *PaWOX3i* lines. (a) Percentage of aberrant cotyledons (folded + forked cotyledons) in control and *PaWOX3i* lines. Percentages are means ± SE of three biological replicates. In each replicate > 150 cotyledons per line were analysed. Different letters indicate significant differences in the frequency of aberrant cotyledons among lines (Student–Newman–Keuls test, α = 0.05). (b–d) Cotyledon phenotypes in 6‐wk‐old plants: (b) normal cotyledons; (c) folded cotyledons; (d) forked cotyledons (two cotyledons fused). (e) Cross‐section of a normal cotyledon (left) and a folded cotyledon (right). Note the lateral outgrowth in the normal cotyledon and the lack of lateral outgrowth in the folded cotyledon (black arrowheads). (f) Needle phenotype in 3‐month‐old plants: a needle with normal phenotype from an U‐control plant (upper image), and three aberrant needles from *PaWOX3i* lines. Note the different shape and the reduction in the number of sawtooth hairs (red arrowheads) in aberrant needles from *PaWOX3i* lines. Bars, 1 mm.

More than 97% of the 150 analysed cotyledons from 6‐wk‐old control plants had a normal flattened morphology (Fig. 8a,b). By contrast up to 33% of the cotyledons from plants from the *PaWOX3i* lines had an aberrant morphology. Two different aberrant morphologies were observed: cotyledons with a more rounded shape which were folded in the middle‐apical part (Fig. 8c) and, at a low frequency (< 1%), forked cotyledons, which were never observed in control plants (Fig. 8d). In contrast to the lateral outgrowth observed in cross‐sectioned normal cotyledons, folded cotyledons lacked the lateral outgrowth resulting in a more rounded shape (Fig. 8e). Forked cotyledons had two vascular bundles, suggesting that the phenotype was caused by fusion of two cotyledons (Fig. S4). The frequency of aberrant cotyledons was significantly higher in *PaWOX3i* lines than in controls (Fig. 8a). Furthermore, the *PaWOX3i* lines (*PaWOX3i.3*,* PaWOX3i.13*) with the strongest downregulation of *PaWOX3* (Fig. 6) had the highest frequency of aberrant cotyledons. A statistically significant inverse correlation was found between the frequency of abnormal cotyledons and the mRNA levels of *PaWOX3* (Pearson correlation coefficient = −0.91, *P *=* *0.012). Similar frequencies of aberrant cotyledons were found after 12 wk on germination medium (Fig. S5).

Needles from *PaWOX3i* plants grown for 12 wk in the glasshouse had an aberrant morphology (Fig. 8f). The needles were more rounded in shape compared with the flattened needles in the corresponding control plants. In addition, the number of sawtooth hairs was drastically reduced in needles from *PaWOX3i* plants.

### *PaWOX3* is important for root elongation

Radicle emergence (germination frequency) was evaluated in 50 embryos from each control and *PaWOX3i* line after 4 wk on germination medium. More than 90% of the control embryos germinated. No significant differences in germination frequency were found between embryos from control and *PaWOX3i* lines (Fig. S6).

**Figure 9:**
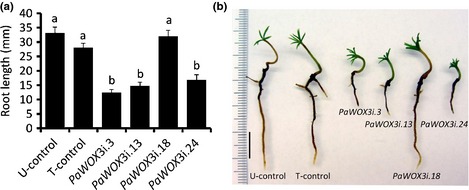
Root development in Norway spruce *(Picea abies)* plants from control and *PaWOX3i* lines. (a) Average root length in plants from control and *PaWOX3i* lines after 16 wk on germination medium. Presented root lengths are means ± SE of three biological replicates. In each replicate 25 plants per line were analysed. Different letters indicate significant differences in the root length among lines (Kruskal‐Wallis One Way Analysis of Variance on Ranks, α = 0.05). (b) Representative plants from control and *PaWOX3i* lines after 16 wk on germination medium. Bar, 1 cm.

Root length and number of lateral roots were estimated after 16 wk on germination medium. The average root length in plants from all *PaWOX3i* lines except from line *PaWOX3i.18*, which showed the highest mRNA level of *PaWOX3* out of the four lines examined, was significantly shorter than in control plants (Fig. 9). A detailed observation of the root tips revealed that the differentiation of root hairs was disturbed in *PaWOX3i.3*,* PaWOX3i.13* and *PaWOX3i.24* plants, and the RAM region showed a reduced size (Fig. S7). The number of lateral roots per plant varied from 0 to > 10 in all lines, with an average of 2.5 lateral roots per plants. The number of lateral roots per plant showed a large variation between plants of all genotypes, and no statistically significant differences were found between the controls and the *PaWOX3i* lines.

## Discussion

In this work, we present direct genetic evidence for the requirement of *PaWOX3* for normal cotyledon and needle development in Norway spruce. Germinated embryos and plants with reduced transcript abundance of *PaWOX3* showed aberrant cotyledon and needle morphology: specifically, defects in lateral margin outgrowth and in the development of sawtooth hairs along the needle lateral margins. These defects are consistent with the expression pattern of *PaWOX3*. The expression of the gene was detected in mature embryos at the base of each cotyledon at the junction between neighbouring cotyledons, in the domain between the adaxial and abaxial side of young developing cotyledons, and in later stages in the basal part of the cotyledons and in developing sawtooth hairs. Thus, the expression is highly specific to those organs and tissues that are phenotypically altered in the transgenic embryos and regenerated plants. We conclude that *PaWOX3* in Norway spruce acts to promote lateral margin development and expansion in cotyledons and needles, thereby establishing the ad/abaxial polarity of these organs. The similarities in expression patterns in needle primordia between *PaWOX3* and its orthologues in the gymnosperms *Ginkgo biloba*,* Gnetum gnemon* and *Pinus sylvestris* (Nardmann & Werr, 2013), suggest that the expression pattern and functions of *PaWOX3*‐related genes might be shared between all gymnosperms.

From an evolutionary perspective, the function and expression pattern of *PaWOX3* in lateral organs is interesting because the similarities to the corresponding properties of one of the orthologues of *PaWOX3* in angiosperms (*PRS*) are quite extensive. In Arabidopsis embryos, *PRS* is expressed at the margins of cotyledon primordia (Haecker *et al*., 2004) and later at the apices and the lateral margins of cotyledons, defining a border between the ad‐ and abaxial sides of the cotyledons (Nardmann *et al*., 2004). In leaves, *PRS* is expressed specifically in lateral regions of young primordia (Matsumoto & Okada, 2001). The maize *PRS* orthologue *NS* shows a similar expression pattern (Matsumoto & Okada, 2001). Despite morphological differences, available data on the phenotypic alterations associated with mutations in angiosperm orthologues of *PaWOX3*, and our data on Norway spruce, suggest similarities in gene function across seed plants. Arabidopsis *PRS* and maize *NS1/NS2* perform a similar function in the recruitment of founder cells from lateral domains of shoot meristems that form lateral and marginal regions of leaves and flower organs (Scanlon *et al*., 1996; Matsumoto & Okada, 2001; Nardmann *et al*., 2004; Shimizu *et al*., 2009). Furthermore, leaves and petals in Arabidopsis double mutants for *PRS* and *WOX1* are narrower and more curled than those of the wild‐type or either single mutant, suggesting that *PRS* and *WOX1* act redundantly to regulate blade outgrowth in leaves (Nakata *et al*., 2012). Similar alterations in leaf development have been reported to occur in the maize *ns1/ns2* double mutant (Nardmann *et al*., 2004), and in the rice *nal2/nal3* double mutant (Cho *et al*., 2013). Thus, the phenotypic consequences of *PaWOX3* knockdown in Norway spruce resemble those observed in mutants for the orthologous genes in angiosperms. Both in Norway spruce and angiosperm models, needles/leaves and cotyledons are narrower and curled or folded, and lateral margin development is impaired, indicating that *WOX3* is instrumental for lateral margin cell growth and development.

In addition to the shoot phenotype, root length was also affected negatively by *PaWOX3* downregulation in three out of the four *PaWOX3* RNAi lines studied. The *PaWOX3* function in the root is consistent with the expression of the gene in this organ. In Arabidopsis and maize the *WOX3*‐related genes appear to lack a specific function in the root, consistent with their absence of expression in the root, whereas in rice *WOX3* is expressed in roots as well as in leaf blades (Cho *et al*., 2013). In rice *nal2/nal3* double mutant plants the number of lateral roots is significantly reduced although no effect on root length was reported. In our experiments on Norway spruce, no significant differences in the number of lateral roots were observed between control and *PaWOX3i* plants, suggesting that the function of *PaWOX3* in the root might be different from that of its rice orthologue. Our attempts to determine the spatial pattern of *PaWOX3* expression in the root have as yet been unsuccessful. Our β*‐*glucuronidase (GUS) reporter gene expression data from roots are inconsistent with RNA expression data, and, therefore, presumably does not represent the transcriptional activity of the gene in this organ. Possibly, the 2.025‐bp promoter fragment used in our construct is insufficient to promote adequate transcription specifically in the root. In addition, our *in situ* hybridization protocol for conifer material has so far not been successfully applied to root tissue. Therefore, future studies will be necessary to elucidate the expression pattern and function of *PaWOX3* in roots.

The simplest explanation for the similarities and differences found between the *WOX3*‐related genes in angiosperms and Norway spruce, is that the genes derive from one original *WOX3*‐like gene present in the last common ancestor of angiosperms and gymnosperms. The functional properties and expression patterns of *PaWOX3* and its orthologues in angiosperms indicate that *WOX3* gene function in the developing shoot is largely conserved between gymnosperms and angiosperms. This gene would have functioned in the regulation of lateral expansion and development, and the distinction of ad/abaxiality in lateral organs. The role of the conifer *WOX3* gene in root development might also be a reflection of the function of the ancestral gene in this context. If so, the root function of *WOX3* would have been lost in the angiosperm lineage. A similar loss of root activity in a gene lineage originating from an ancestral gene with activity in both shoot and root meristems has been proposed for the angiosperm *WUS* gene lineage (Nardmann *et al*., 2009). Alternatively, the *WOX3* function in root development might be a novelty, gained in the lineage leading to the conifers, after the split from the angiosperms.

In conclusion, our results suggest that the last common ancestor of the extant gymno‐ and angiosperms contained a *WOX3* gene ancestor associated with margin outgrowth in lateral organs. In addition, we suggest a role for *PaWOX3* in root development, which might be specific for gymnosperms.

## Supporting information

Please note: Wiley Blackwell are not responsible for the content or functionality of any supporting information supplied by the authors. Any queries (other than missing material) should be directed to the *New Phytologist* Central Office.

**Fig. S1** Schematic illustration of the CDS of *PaWOX3* and RNAi construct.
**Fig. S2** Developmental pathways of normal and *PaWOX3i* embryos.
**Fig. S3** Number of cotyledons per embryo in control (U‐control and T‐control) and *PaWOX3i* lines.
**Fig. S4** Cross‐section of an aberrant forked cotyledon.
**Fig. S5** Percentage of aberrant cotyledons (fold + fork cotyledons) in plants from control and *PaWOX3i* lines after 12 wk on germination medium.
**Fig. S6** Germination frequency (percentage of embryos with radicle elongation) after 4 wk on germination medium.
**Fig. S7** Root tips from control and *PaWOX3i* plants.
**Table S1** Primer sequences used for qRT‐PCR analysis, RNAi and ISH
**Notes S1** GUS staining in mature embryos.
