# Supplementary material for: The WUSCHEL‐RELATED HOMEOBOX 3 gene PaWOX3 regulates lateral organ formation in Norway spruce
Source: New Phytol. 2015 Jun 25;208(4):1078–88. doi: 10.1111/nph.13536 (PMC5034847; doi:10.1111/nph.13536)
Supplement: Supplementary file 1 — Fig. S1 Schematic illustration of the CDS of PaWOX3 and RNAi construct. Fig. S2 Developmental pathways of normal and PaWOX3i embryos. Fig. S3 Number of cotyledons per embryo in control (U‐control and T‐control) and PaWOX3i lines. Fig. S4 Cross‐section of an aberrant forked cotyledon. Fig. S5 Percentage of aberrant cotyledons (fold + fork cotyledons) in plants from control and PaWOX3i lines after 12 wk on germination medium. Fig. S6 Germination frequency (percentage of embryos with radicle elongation) after 4 wk on germination medium. Fig. S7 Root tips from control and PaWOX3i plants. Table S1 Primer sequences used for qRT‐PCR analysis, RNAi and ISH Notes S1 GUS staining in mature embryos. [file NPH-208-1078-s001.pdf]

***New Phytologist* Supporting Information**

Article title: The *WUSCHEL-RELATED HOMEOBOX 3* gene *PaWOX3* regulates lateral organ formation in Norway spruce

Authors: José M. Alvarez, Joel Sohlberg, Peter Engström, Tianqing Zhu, Marie Englund, Panagiotis N. Moschou and Sara von Arnold

Article acceptance date: 29 May 2015

The following Supporting Information is available for this article:

Table S1 Primer sequences used for qRT-PCR analysis, RNAi and ISH

Notes S1 GUS staining in mature embryos

Fig. S1 Schematic illustration of the CDS of *PaWOX3* and RNAi construct

Fig. S2 Developmental pathways of normal and *PaWOX3i* embryos

Fig. S3 Number of cotyledons per embryo in control (U-control and T-control) and *PaWOX3i* lines

Fig. S4 Cross-section of an aberrant forked cotyledon

Fig. S5 Percentage of aberrant cotyledons (fold + fork cotyledons) in plants from control and *PaWOX3i* lines after 12 weeks on germination medium

Fig. S6 Germination frequency (percentage of embryos with radicle elongation) after 4 weeks on germination medium

Fig. S7 Root tips from control and *PaWOX3i* plants

Table S1 Primer sequences used for qRT-PCR analysis, RNAi and ISH

	Name	Sequence
qRT-PCR	*PaWOX3* F	5’-ACGAAAACCAAGAGCTTCGCCA-3’
	PaWOX3 R	5’-CGGGCTCTGGAAACCACGCC-3’
	PaEF1 F	5’-CACCTTGGGAGTGAAGCAAATG-3’
	PaEF1 R	5’-GGGAGTAGTGGCATCCATCTTG-3’
RNAi	*PaWOX3* 1-F	5’-GAATTCGGATCCACTGGTTTCAGAATCACAAAGCTCG-3’
	PaWOX3 2-F	5’-GAATTCGGATCCATCACAGCACATCTGGCTTTGTATG-3’
	PaWOX3 1-R	5’-**CACC**CTATAAGCCACTGCATGTTGGAAAC-3’
	PaWOX3 2-R	5’-CTATAAGCCACTGCATGTTGGAAAC-3’
ISH	*PaWOX3* F	5’-ACAATGGCTGGAGGCTTAG-3’
	PaWOX3 R	5’-TTCCAGAGTTTTGAGAGGC-3’

The underlined sequences show enzyme digestion sites. Sequence in bold designates the TOPO-compatible sequence. qRT-PCR: quantitative real-time PCR; RNAi: RNA interference; 1: fragment 1; 2: fragment 2; ISH: *in situ* hybridization; F: forward; R: reverse.

Notes S1 GUS staining in mature embryos. Strong GUS staining was detected in the basal part of mature embryos within 6 hours incubation in GUS solution and showed a rapid diffusion into the surrounding tissues. The upper part of the embryo showed no GUS staining after 24h incubation (see figure NS1). In order to determine if the strong GUS activity in the basal part of the embryos really reflected a high expression of *PaWOX3* the embryos were divided into two parts and were designated as upper part (including SAM and cotyledons) and basal part (including RAM and root cap). The mRNA abundance of *PaWOX3* in the upper and basal part of the embryos was analyzed by qRT-PCR. The mRNA level of *PaWOX3* was very low in the basal part (see figure NS1). It is unclear why the GUS staining and the steady-state mRNA levels of *PaWOX3* do not spatially coincide. However, since we have observed similar strong GUS staining in the basal part of mature embryos using different promoters we assume that the GUS signal in the basal part of the embryo is unspecific. This discrepancy might indicate that the GUS expression in the basal part of the embryo is not reliable. Intrinsic GUS activity in mature embryos should be disregarded as no GUS staining could be detected in the U-control. To avoid GUS diffusion, the basal part of the embryo was removed and the incubation time in GUS solution was extended to 72 h, which made it possible to detect GUS signal from the upper part of the embryo.

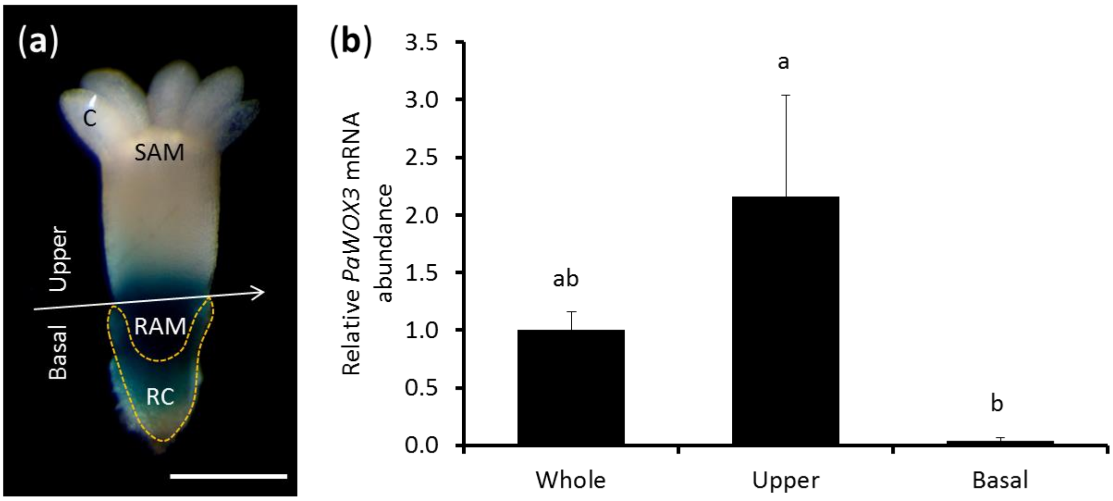

Figure NS1. Expression of *PaWOX3* in mature embryos. (a) GUS staining pattern in a longitudinally sectioned embryo after 24 h incubation in GUS solution. Note the strong GUS signal in the basal part and diffusion of the GUS signal to the surrounding tissues. White arrow indicates the excision point for the qRT-PCR analysis. C: cotyledon; SAM: shoot apical meristem; RAM: root apical meristem; RC: root cap. (b) Relative *PaWOX3* mRNA abundance analyzed by qRT-PCR in the whole embryo, upper part and basal part. The mRNA abundances are relative to the transcript level in the whole embryo and normalized

against *PaEF1*. The mRNA abundances are means ± SE of three biological replicates. Different letters indicate significant differences in the relative *PaWOX3* mRNA abundance among the different embryos parts (Student-Newman-Keuls test, α=0.05). Scale bar = 1 mm.

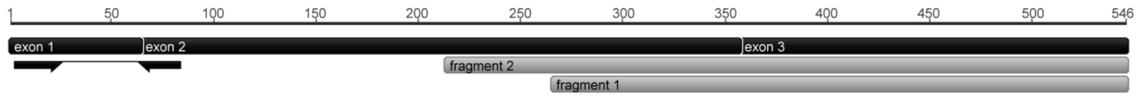

Fig. S1 Schematic illustration of the CDS of *PaWOX3* and RNAi construct. The black boxes represent the gene exons. The grey boxes represent the two fragments used for the RNAi construct. The black arrows represent the primers used for qRT-PCR analysis. Primer sequences are listed in Supplementary Table 1.

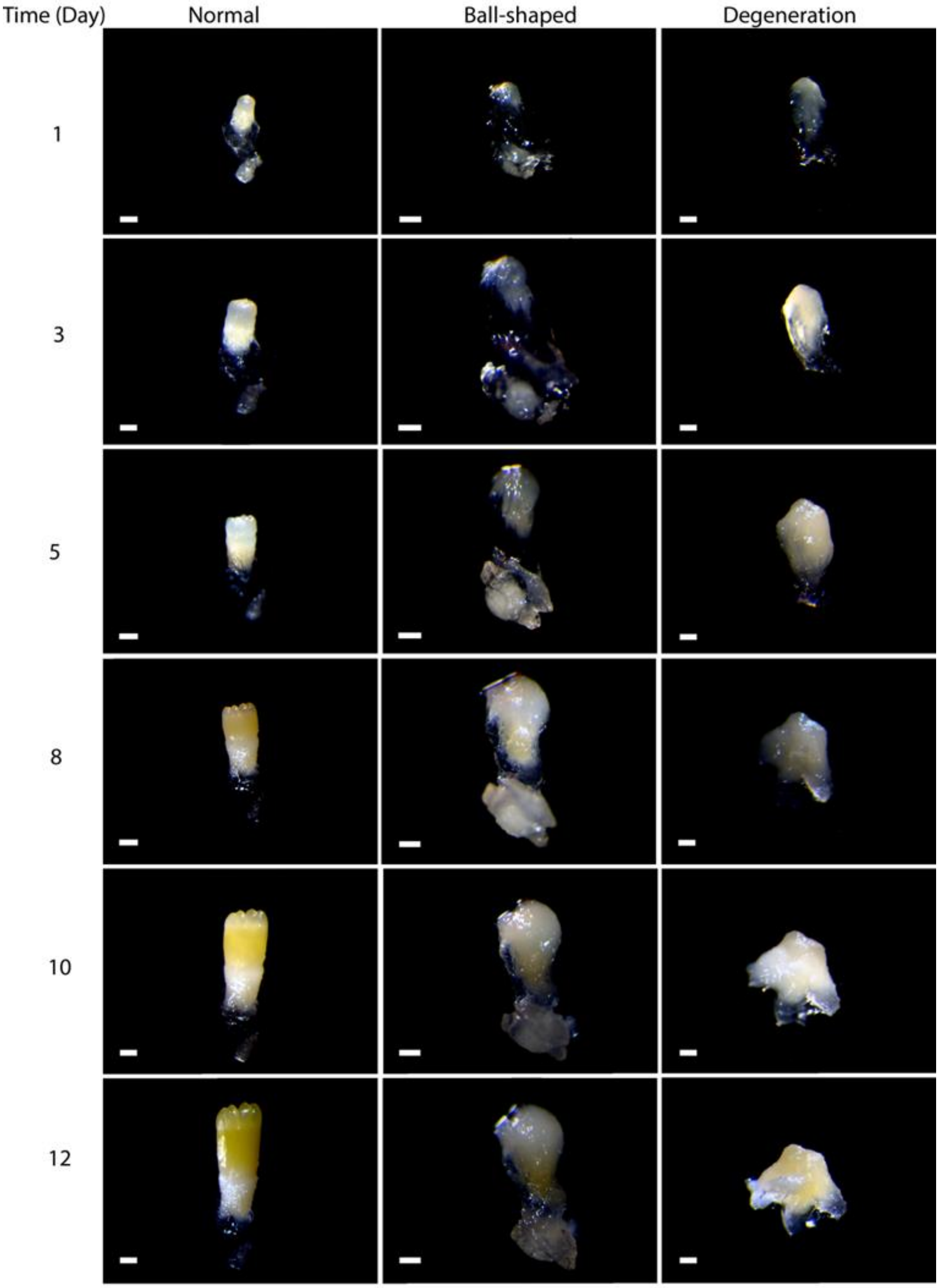

Fig. S2 Developmental pathways of normal and *PaWOX3i* embryos. The development of 50 randomly selected embryos (LE1s) from each line was tracked for 12 days. The LEs were sampled after 2 weeks on maturation medium and transferred to fresh maturation medium. Photos are presented for day 1, 3, 5, 8, 10 and 12. Three developmental pathways were observed in embryos from all lines: i) normal

development (about 80% of the LE1s in the U-control and 65% of the LE1s in all transformed lines); ii) ball-shaped development, in which the embryos lack differentiated cotyledons (about 20% of the LE1s in the U-control and 35% of the LE1s in all transformed lines); and iii) degenerated development, in which embryogenic tissue differentiates from the first selected embryo (1-3% of the LE1s in all lines). Since a similar increase in the frequency of aberrant ball-shaped embryos was observed in all transgenic lines, including the T-control in which the level of *PaWOX3* mRNA was similar as in the U-control, we assume that the aberrant development is a consequence of the transformation itself and that the *PaWOX3* depletion is not critical for the development of MEs. Scale bar = 100µm.

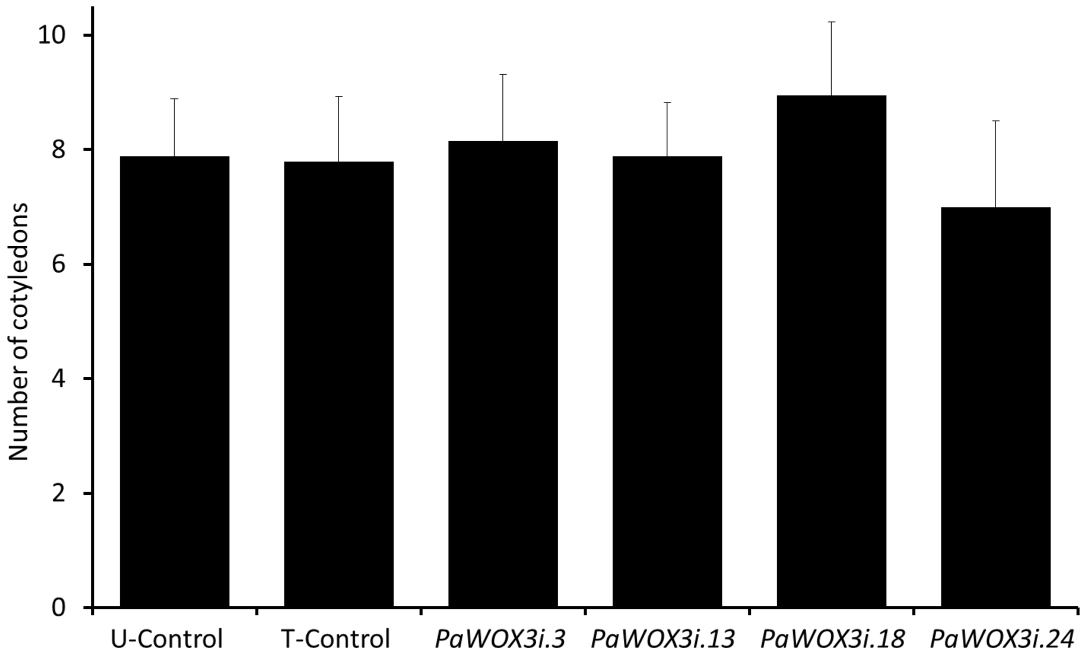

Fig. S3 Number of cotyledons per embryo in control (U-control and T-control) and *PaWOX3i* lines. The number of cotyledons are means ± SE of three biological replicates. In each replicate 25 embryos per line were analyzed.

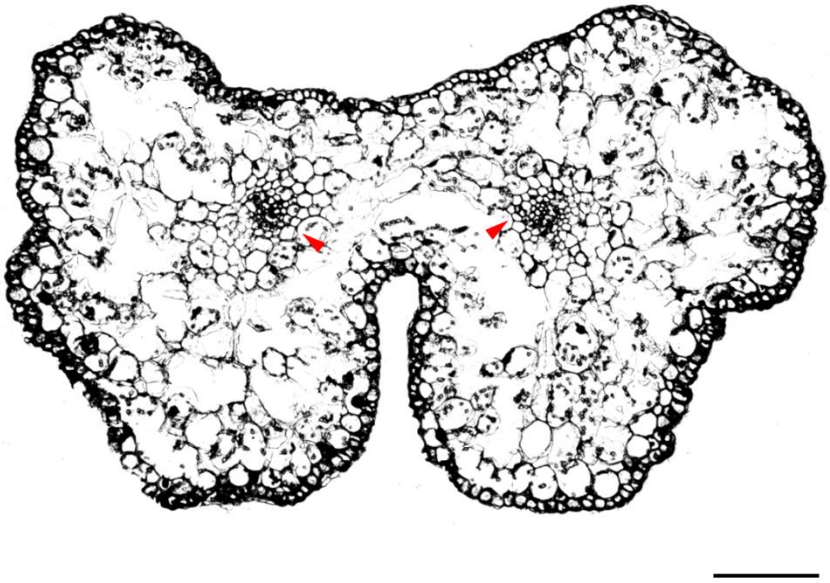

Fig. S4 Cross-section of an aberrant forked cotyledon (Fig.8d). Note the two vascular bundles (red arrowheads). Scale bar = 0.1 mm

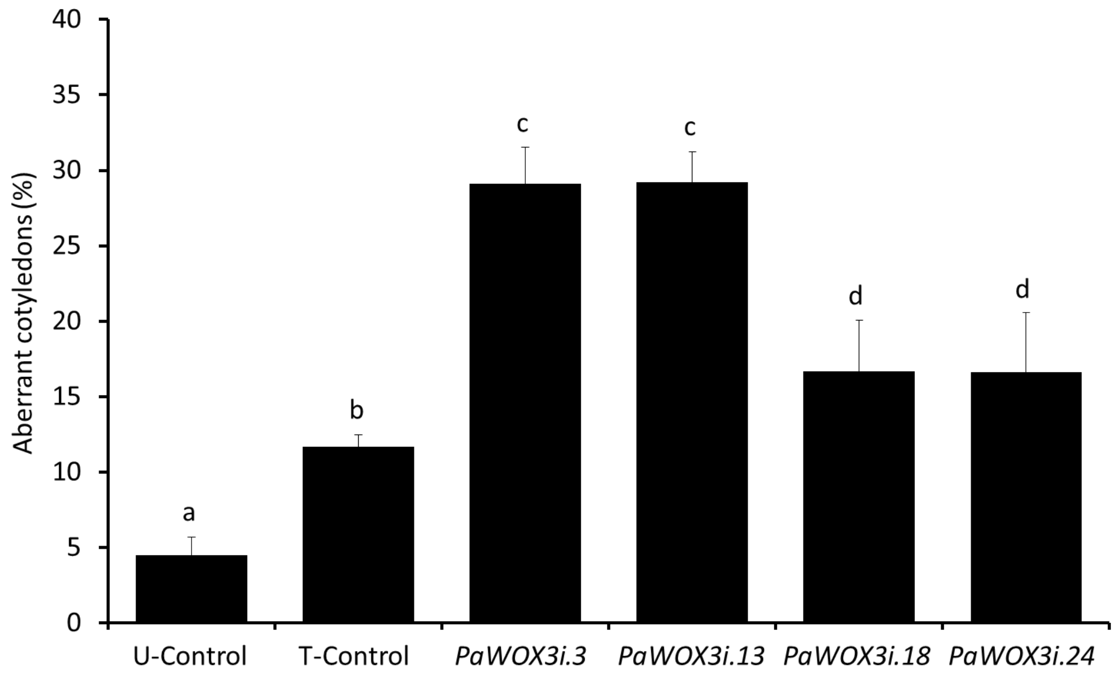

Fig. S5 Percentage of aberrant cotyledons (fold + fork cotyledons) in plants from control and *PaWOX3i* lines after 12 weeks on germination medium. Percentages are means ± SE of three biological replicates. In each replicate 150 cotyledons per line were analyzed. Different letters indicate significant differences in the frequency of aberrant cotyledons among the different lines (Student-Newman-Keuls test, α=0.05).

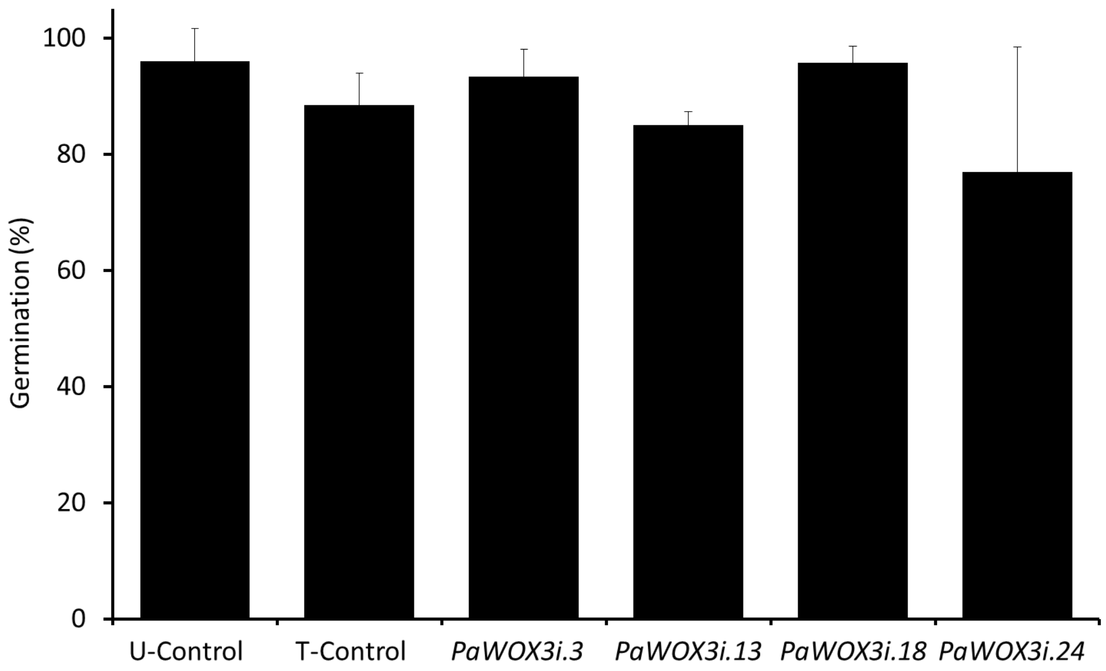

Fig. S6 Germination frequency (percentage of embryos with radicle elongation) after 4 weeks on germination medium. The number of germinated embryos are means ± SE of three biological replicates. In each replicate 50 embryos per line were analyzed.

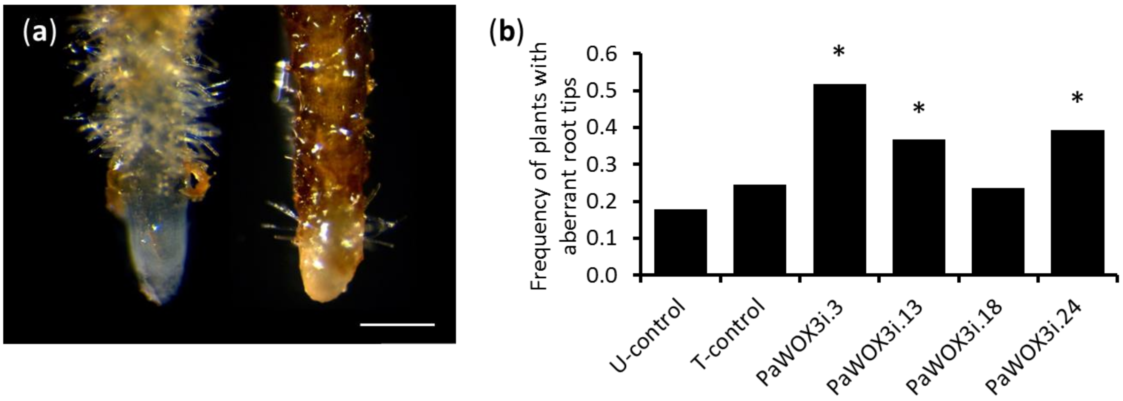

Fig. S7 Root tips from control and *PaWOX3i* plants. (a) Normal root tip with root hairs (left) and aberrant root tip where differentiation of root hairs is disturbed (right). Scale bar = 0.5 mm. (b) Frequency of plants with aberrant roots in control (U-control and T-control) and *PaWOX3i* lines. Fifty roots per line were examined after 16 weeks on germination medium. Asterisks indicate significant differences between control and *PaWOX3i* lines (χ^2 test, α=0.075).
